# A Multilingual Integrative Review of Health Literacy in Former Soviet Union, Russian-Speaking Immigrants

**DOI:** 10.3390/ijerph18020657

**Published:** 2021-01-14

**Authors:** Uliana Kostareva, Cheryl L. Albright, Eva-Maria Berens, Patricia Polansky, Deborah E. Kadish, Luba L. Ivanov, Tetine L. Sentell

**Affiliations:** 1 School of Nursing and Dental Hygiene, University of Hawai’i at Mānoa, Honolulu, HI 96822, USA; cherylal@hawaii.edu; 2 Thompson School of Social Work and Public Health, University of Hawai’i at Mānoa, Honolulu, HI 96822, USA; tsentell@hawaii.edu; 3 Interdisciplinary Centre for Health Literacy Research, Bielefeld University, 33615 Bielefeld, Germany; eva-maria.berens@uni-bielefeld.de; 4 Hamilton Library Russian Bibliographer, University of Hawai’i at Mānoa, Honolulu, HI 96822, USA; polansky@hawaii.edu; 5 Center for Evaluation of Health Promotion Interventions, Faculty of Social Welfare and Health Sciences, University of Haifa, Haifa 31000, Israel; d.kadish@gmail.com; 6 Chamberlain College of Nursing, Chamberlain University, Downers Grove, IL 60515, USA; LIvanov@chamberlain.edu

**Keywords:** health literacy, healthcare system, immigrant/migrant/refugee, cultural and linguistic care practices, patient education

## Abstract

Large diasporas of former Soviet Union (FSU) immigrants are found in the USA, Germany, and Israel. To synthesize evidence, identify limitations, and propose future directions we conducted an integrative review on the health literacy of FSU immigrants, migrants, or refugees in four languages. Following integrative review and PRISMA guidelines, we searched four databases in English and performed supplementary searches in Russian, German, and Hebrew to identify qualitative and quantitative studies on FSU immigrants and health literacy. Six articles met inclusion criteria in English and one in German; the majority were published in the last five years. Only two articles measured health literacy of FSU immigrants, which was lower than the general population. Four articles were about immigrants with a mean age ≥50 years. All articles stressed the value of translated, culturally relevant health information. The health literacy of FSU immigrants is understudied, despite clear needs. Future research should include assessments of FSU immigrants’ health literacy and include diverse (e.g., age, gender) yet well-defined populations to determine both barriers and facilitators to their health literacy. This review, an example of a multilingual search, provided a comprehensive understanding of existing literature and is a useful approach for global health literacy research.

## 1. Introduction

Internationally, health literacy is an important predictor of health [1]. Several aspects of health literacy are critical to health outcomes, including organizational and personal health literacy [2]. The definition of personal health literacy has been recently updated by the American Healthy People 2030 goals as “the degree to which individuals have the ability to find, understand, and use information and services to inform health-related decisions and actions for themselves and others” [2].

Personal health literacy levels have been attributed to social determinants [3,4,5]. Limited health literacy has been linked to outcomes including poorer health status, increased risk of hospitalization, and higher health care expenses [6,7,8,9,10,11]. Individual factors such as migration status, language proficiency, and socioeconomic factors across multiple countries also play a role in health literacy. For example, in the United States of America (USA), low levels of health literacy were more prevalent among recent immigrants, foreign-born, and those with limited English proficiency [12,13,14,15]. In Germany, health literacy was found to be lower among people with a migration history, of lower social status, and older age [16,17]. In Israel, lower health literacy was reported more frequently among people with lower education, income, and among minority communities [18]. In addition, immigrants, being unfamiliar with the healthcare systems of their host countries, may have difficulty utilizing healthcare services effectively [19].

Importantly, in the time of the COVID-19 pandemic, during which international movement has contributed to the spread of the disease and challenged containment and management of outbreaks, there is a clear need for immigrant-friendly health information that is available in multiple languages, accessible, accurate, and easy to understand and act upon [20,21]. This need also relates to the information on prevention strategies such as vaccination [22,23,24]. During the pandemic, efforts to increase public and individual health literacy have become a critical component of public health [25,26].

Thus, our review investigated the health literacy of one large, critical population of international migrants—people from the former Soviet Union (FSU) region. International migrants are people who live outside of their native country’s borders [27]. “Immigrant” typically refers to a person who moved across an international border with the intent of permanent relocation [27]. Other terminologies are also used for people who relocate outside of their native country permanently or temporarily (e.g., refugees, migrants), but in this article, we use the word “immigrant” generally to include migrants and refugees.

Russian-speaking FSU immigrants are a globally important population to understand. Geographically, the FSU region consists of 15 independent countries (Armenia, Azerbaijan, Belarus, Estonia, Georgia, Kazakhstan, Kyrgyzstan, Latvia, Lithuania, Moldova, Russian Federation (Russia), Tajikistan, Turkmenistan, Ukraine, Uzbekistan), also referred to collectively as former Soviet republics [28]. With the Soviet Union’s dissolution in 1991, many people moved between the former republics as well as internationally. For example, an estimated 25–30 million Russians live outside of Russia, many emigrated to the USA, Germany, and Israel [28,29,30]. The top three former Soviet republics from which FSU immigrants originate are Russia (10.5 million), Ukraine (5.9 million), and Kazakhstan (4 million) [31]. Russia alone is the fourth in the world in the size of diaspora populations after India (17.5 million), Mexico (11.8 million), and China (10.7 million) [31]. A substantial amount of literature describes FSU immigrants’ background, migration reasons, and processes. These contextual factors were recently summarized in relation to health literacy, particularly noting the importance of access to and utilization of preventative and medical care in their host countries and differences across healthcare systems [32].

In the 2010s, Russian-speakers appeared to continue to leave their native countries in growing numbers, representing a second modern wave of emigration—the wave of emigration in the 1990s being the first [33,34,35,36,37]. Although Russian speakers have been moving across countries for centuries, this integrative review focuses on FSU immigrants who emigrated after the collapse of the Soviet Union. Also, we specifically focus on the Russian-speaking FSU immigrants because Russian is the most common language spoken across 15 FSU republics [28,38]. This description allows the most inclusive but specific approach that is based on the shared linguistic and historical background rather than an ethnic, national, or religious distinction (e.g., Ukrainian, Jewish).

FSU immigrants are particularly important to understand in the USA, Germany, and Israel—the most common countries of their destination. Approximately 3–5 million people in the USA claim ancestry from the FSU region, which includes both recent immigrants and descendants of immigrants [29,39]. Unverified estimates suggest that up to 7 million people in the USA speak Russian while the official estimate is closer to about 1 million [40,41]. In Germany, there are 3.5 million Russian-speaking FSU first- and second-generation immigrants [42]. In Israel, there are almost 1.3 million FSU immigrants, representing more than a third of all Israeli immigrants [43].

To date, few research or survey investigators describe the health literacy of Russian-speaking FSU immigrants despite their substantial prevalence among international migrants. For example, two recent systematic reviews that examined the health literacy of immigrants in the European Union (EU) and health literacy interventions for immigrants across countries did not contain articles describing FSU immigrants [44,45]. To inform effective health care delivery, disease prevention, and health promotion practices and policies in a culturally and linguistically appropriate manner, it is important to understand health literacy in immigrants, including FSU immigrants. Given the importance of this topic, our interdisciplinary international team performed a multilingual, in-depth literature search to inquire into publications that addressed the growing number of FSU immigrants globally. Our review’s primary goal was to identify publications addressing the health literacy of FSU immigrants across the USA, Germany, and Israel, and to synthesize the results to identify evidence, research gaps and limitations, implications for practice, and to pose future research questions spanning international borders.

In addition to health literacy in general, we were also interested in existing research specifically on health insurance literacy, a sub-concept within health literacy. Health insurance literacy is defined as “the degree to which individuals have the knowledge, ability, and confidence to find and evaluate information about health plans, select the best plan for their own (or their family’s) financial and health circumstances, and use the plan once enrolled” [46]. This topic is particularly important in the USA, given the complexity of its health insurance system, although Germany and Israel also have private health insurance options that go beyond universal health coverage [32,47,48]. Unfamiliar healthcare systems could be particularly confusing to FSU immigrants whose health care experiences originate from the Soviet and post-Soviet healthcare systems [32]. The identification of articles on this topic was an additional study goal.

## 2. Materials and Methods

We used the integrative review approach to be inclusive of literature with diverse methodologies [49]. Following the integrative review guidelines, after identification of the problem, we performed a literature search in four languages, evaluated selected articles, analyzed data, and provided implications, for practice, research, and policy [49]. Article evaluation included two components: (1) characteristics of populations included in the review were defined (e.g., age, education, country of origin, year migrated; not including publicly available data); and, (2) implications for practice, research, or policy discussed. Publications that had one component were labeled 1, publications with both components were labeled 2. We chose these as the most informative for our understanding of health literacy in FSU immigrants and next steps. In addition to integrative review articles, we added a section on relevant articles that did not meet our inclusion criteria but were still informative to our overall goal of understanding health literacy in FSU immigrants.

### 2.1. Inclusion and Exclusion Criteria

To be included, an article had to explicitly focus on health literacy (or health insurance literacy) and have a sample that included adult Russian-speaking FSU immigrants, or migrants, or refugees. There were no limitations on the time frame or study design. We included empirical, theoretical, qualitative, and quantitative studies. We excluded papers not published in a peer-reviewed journal and scale development studies. If health literacy concerning Russian-speaking FSU immigrants was not a clear research topic of the article, it was excluded. For instance, we excluded publications that mentioned a sample of a few FSU immigrants or Eastern Europeans among many other immigrant groups but did not provide a description of FSU immigrants or compare them directly with other groups (native-born or other immigrants). Our goal was to highlight FSU-specific insights, not challenges among heterogeneous immigrant groups in general. Table 1 summarizes the inclusion and exclusion criteria.

### 2.2. English Search

We searched the English language literature following PRISMA (Preferred Reporting Items for Systematic Reviews and Meta-Analyses) guidelines using four databases: PubMed Medline, CINAHL (The Cumulative Index to Nursing and Allied Health Literature) via EBSCO host, Web of Science, and PsycNET [50]. Our search did not limit dates in the past and included articles published up to 16 September 2020. Search terms included: Russia(n) OR Soviet; AND, emigrants or immigrants [Mesh] for PubMed or immigrant OR migrant OR refugee; AND, health literacy as an exact phrase. In addition, in PubMed, each of the names of the 15 FSU republics, now independent countries, were searched separately (e.g., “health literacy” AND Kazakhstan) to identify relevant articles, which yielded no additional articles beyond the more general search (Russia(n) OR Soviet). We also included health insurance literacy as an exact phrase in our search and searched up to 14 October 2020. Additionally, we conducted secondary searches of the reference list reported in relevant articles. Two independent reviewers (U.K., T.L.S.) performed all screening separately and met for consensus. A third reviewer (C.L.A) was available to resolve disagreements, but none occurred.

### 2.3. Russian, German, and Hebrew Searches

We performed supplemental searches in additional languages: Russian, German, and Hebrew. This was done to increase the depth and comprehension of our understanding of health literacy in FSU immigrants across borders. Considering, that besides the USA, the majority of FSU immigrants that settled in Germany and Israel originate from fully or partially Russian-speaking countries, we felt compelled to examine literature in all relevant languages to understand the overall scope and variety of research efforts and to identify gaps and future opportunities. Specific methods and findings of supplemental searches by each language are summarized in Section 3.2.

## 3. Results

### 3.1. English Language Articles

**Figure 1:**
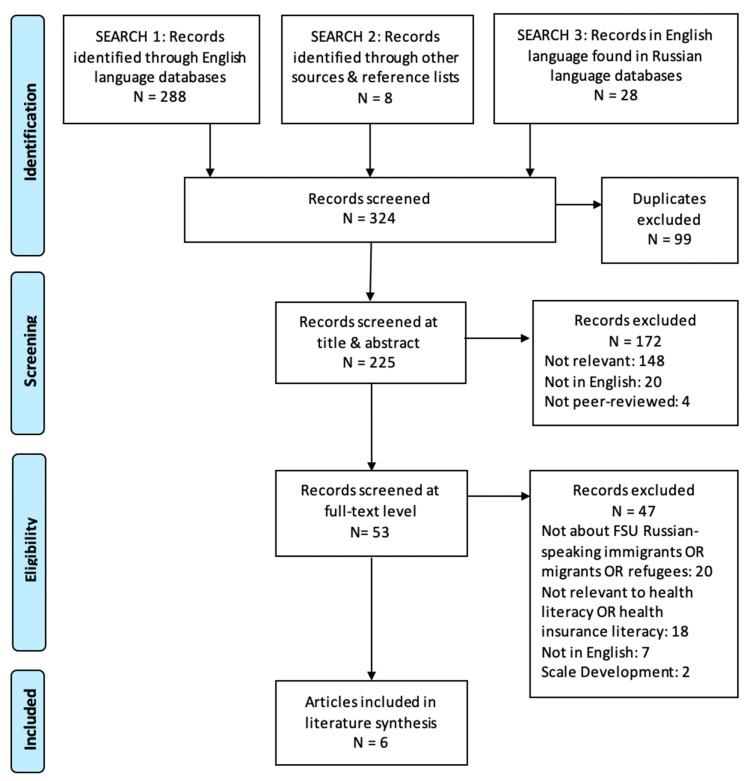
PRISMA flow diagram for English language articles [50].

We found 324 articles in English (see Figure 1) about the health literacy of FSU immigrants. We were not able to identify any articles that specifically addressed both health insurance literacy and Russian-speaking FSU immigrants. After excluding duplicates, 225 articles were screened at the title and abstract level and 53 at the full-text level with six articles included in the final literature synthesis.

### 3.2. Russian, German, Hebrew Languages Articles

Supplemental searches in three other languages yielded one article in German. Search strategies were somewhat different from the English language search and thus, were not included in the PRISMA flow diagram. Table 2 highlights the main findings and search strategies.

### 3.3. Summary of Studies

The seven included studies were diverse in design and health literacy focus, representing public health, nursing, medicine, social work, and psychology disciplines (Table 3). Most studies (n = 3) were conducted in the USA; two were conducted in Israel; one was from Germany; and, one was international (five countries). Identified articles were recent; for example, the oldest was published in 2009 (n = 1), and the most recent in 2020 (n = 2). Synthesized articles addressed FSU immigrants’ health literacy from both personal and organizational perspectives. Organizational health literacy is defined as “the degree to which organizations equitably enable individuals to find, understand, and use information and services” to make and act upon health decisions [2].

#### 3.3.1. Assessment of Health Literacy in FSU Immigrants

Only two articles directly measured health literacy (general and mental) in FSU immigrants, and both were conducted in Israel [18,53]. Levin-Zamir et al. (2016) validated a 16-question version of the Health Literacy Survey (HLS-EU), a Likert style four-option answer questions originally developed and performed among countries of the European Union, on a national sample that included FSU immigrants [5,18]. The use of a validated international tool was chosen to allow future comparability of results [18]. Out of the maximum score of 16, a score above 13 implied adequate health literacy level; the results for Arabs (12.7) and FSU immigrants (12.9) indicated “problematic” health literacy, while long-term Israeli Jews (13.5) demonstrated “likely sufficient” health literacy [18]. Nakash et al. (2020) assessed mental health literacy using the Mental Health Literacy Scale (MHLS), a four-question Likert style scale with five-option response categories, among older adults who were pre-screened for cognitive impairment [53]. Besides mental health literacy assessment, the authors were particularly interested in the relationship between mental health literacy and emotional distress [53]. FSU immigrants scored significantly lower on MHLS than Israeli born Jews (13.3 vs. 15.5) and demonstrated significantly higher emotional distress (9.1 vs. 5.2) [53]. Emotional distress was moderated by immigration status such that greater knowledge of how to search for health information (higher MHLS) showed no relationship with FSU immigrants’ emotional distress, unlike among the native Israelis [53]. Also, despite reporting to have more years of education than Israeli-born Jews, FSU immigrants had lower income [53].

The other articles used both qualitative and quantitative methods but did not measure health literacy. For example, one article did not measure health literacy specifically, but was designed to improve older FSU immigrants’ health literacy through customized education materials [54]. Another article identified health literacy among FSU immigrants to be an important topic and noted that future research would benefit from a systematized assessment of FSU immigrants’ health literacy, development of health literacy model for immigrants, and comparison of immigrants’ health literacy across countries to inform management strategies [32].

#### 3.3.2. Characteristics of FSU Immigrants

Well-defined sample characteristics are important in research; in our review, they varied based on the study design and goals. The multi-country scoping review by Kostareva et al. (2020) provided an overview of FSU immigrants’ sociodemographic data, historical background, migration context and included a table with publicly available data with characteristics such as highest level of education and host country’s language proficiency across the USA, Germany, and Israel [32]. Four of the six English language publications mentioned age as a factor. Two specifically focused on the older Russian-speaking FSU immigrants [53,54]. Two others had samples with a mean age of 50 and 64 [18,55]. Although both Israeli studies included immigrants who arrived in 1990 or later (last 30 years) and reported gender, marital status, income, and other variables, the article by Levin-Zamir et al. (2016) grouped these characteristics with long-term Israeli Jews and Arabs to represent the country’s population while Nakash et al. (2020) delineated characteristics for FSU immigrants separately from Israeli born Jews [18,53]. Bailey et al. (2012) specifically focused on immigrants with limited English proficiency and a low-income status and included other variables such as years in the USA and level of education in an aggregate table [55]. Three articles did not report age, educational level, country of origin, year migrated, or socioeconomic variables [52,54,56].

#### 3.3.3. Language and Culture

All articles emphasized the value of translated, culturally relevant health information. Three of the seven studies designed, developed, or validated health materials in Russian [54,55,56]. Two articles highlighted the need for culturally adapted and translated materials for effective disease management [54,55]. In Germany, many FSU immigrants, i.e., German resettlers, were proficient in German, but rarely used information and counseling services and reported difficulties processing health information [52]. In the USA, the lack of diabetes-related educational information in Russian prompted Van Son (2014) to design, develop, and test materials that were culturally adapted to the diets of the Russian-speaking immigrants in an effort to improve and empower self-management of diabetes in an underserved community [54]. The materials were then uploaded online for open access and were downloaded internationally almost 2000 times over two years [54]. Sullivan (2009) described a community partnership between immigrants-refugees, a community literacy program, and nursing students, which allowed nursing students to provide customized educational materials using immigrants’ requests for the most relevant health information, which led to active and engaged participation of refugees in health-promoting activities [56].

The provision of culturally and linguistically appropriate care to immigrants can be looked at from personal and organizational health literacy perspectives [57]. The importance and synergy of both were noted in the German article by Horn et al. (2015) that identified a lack of trust toward healthcare system and health providers as a possible contributor to FSU immigrants’ limited health literacy; this finding emphasized the need for providers to preferably speak Russian (e.g., use qualified interpreter) with their FSU immigrant patients to build trust, improve their understanding of health information, and encourage more effective utilization of health services [52]. In light of patient safety, Bailey et al. (2012) in the USA evaluated the efficacy of drug label instructions by conducting a randomized experimental evaluation among low English proficiency adults and compared standard drug instructions to the “ConcordantRx” instructions that were developed using health literacy “best practices” and translated through a community-based approach [55]. Using these new instructions Russian-speaking participants, as well as all other non-English speakers, demonstrated a significantly better understanding of correct dosing, greater ability to take medications appropriately, and were more likely to correctly consolidate their medications [55]. The multi-country scoping review by Kostareva et al. (2020) depicted a landscape of healthcare system-level factors relevant to health literacy, including departed countries (Russia and Kazakhstan) and receiving countries (USA, Germany, Israel) within the Integrated Health Literacy Model [32,58]. The findings of these studies elucidate the importance of organizational health literacy in which an individual’s abilities are supported by organizational efforts.

### 3.4. Other Articles Describing Constructs Related to Health Literacy

In all four languages, many articles did not meet our inclusion criteria but either: described constructs related to health literacy, were relevant to articles included in the synthesis, suggested an intervention, or were useful to our review’s goal to understand health literacy in FSU immigrants. Therefore, below, we summarized highlights of articles we found to be relevant and their findings compelling.

#### 3.4.1. Healthcare System, Access to Care, and Utilization of Health Services

Unfamiliarity with a healthcare system may be a barrier to accessing and effective utilization of health services to support health. In Israel, universal healthcare coverage, which is also the primary means of healthcare in the FSU region, was not effective at decreasing higher perinatal mortality among FSU immigrants, indicating other factors likely played a role in the utilization of preventative and medical services [59]. Another article analyzed patterns of supplemental health insurance use in Israel and found that in the late 1990s, Russian-speaking FSU immigrants were the least likely to have purchased supplemental health insurance when compared to Arabic speakers or long-term Israelis [60]. In the USA, different age groups of FSU immigrant women chose to access medical care based on the pattern of utilization in their countries of origin rather than the host country [61]. An earlier publication found that FSU immigrants in America overutilized healthcare services and expressed high expectations for health care; however, the majority of the people surveyed were refugees and were eligible for a range of public assistance, including subsidized medical services and housing [62]. Affordability and access to medical care are important factors in health literacy, especially in the USA, where the majority of the population has private health insurance, purchased through an employer, individually, or in combination [63]. Although we did not find any articles on health insurance literacy in FSU immigrants, we identified a few articles that looked at health insurance coverage. The lack of health insurance among FSU immigrants in the USA, and likely among other immigrant communities, contributed to the impaired utilization of preventative and medical services [61,64,65,66]. More research is needed to understand factors that influence FSU immigrants’ utilization of health services.

#### 3.4.2. Health Status

While FSU immigrants were significantly more likely to report poor or fair health compared to the native-born white Americans, they also reported less functional limitations and unhealthy behaviors [67]. Meanwhile, in Germany, FSU immigrants’ health status was worse than in the native population and all-cause mortality was significantly higher, especially among men and those who emigrated after 1996 [68,69]. Interestingly, a recently published German report that assessed health literacy among vulnerable populations such as younger immigrants with lower educational attainment and older immigrants presented no data on FSU-immigrants, although Russian was offered as one of the interview languages [70]. In Israel, researchers analyzed several surveys performed in the 1990s either exclusively or in part among Russian-speaking FSU immigrants and found that they reported lower self-rated health, higher rates of chronic illness or disability, lower utilization of healthcare services, including preventative medicine, and generally, lower levels of satisfaction with the healthcare system; yet, they were more satisfied with the range of medications available when compared to long-term Israelis [71].

Mental health is another important health aspect among FSU immigrants. In the USA and Israel, FSU immigrants experienced unaddressed mental health challenges, possibly because of different cultural perceptions toward mental health [72,73]. Older FSU immigrants in the USA with somatization symptoms, who believed these symptoms to be treatable with a medical rather than a mental health approach, were reportedly seeking medical care out of loneliness [62]. On the other hand, in Germany, FSU immigrants did not differ from the native-born Germans in mental health, but providers emphasized difficulties in conveying diagnosis and recommending treatment [74]. In Israel, a telephone survey performed in 1997 found that Russian-speaking FSU immigrants sought out formal and informal mental health services in patterns similar to long-term Israelis [75]. Given the intricacy and interconnectedness of mental health and physical health, a comprehensive health literacy assessment that includes mental health literacy aspects should be considered in future research.

#### 3.4.3. Language and Acculturation

Language proficiency, acculturation, health beliefs, prior experiences, and cultural norms are important factors related to health literacy and health outcomes in immigrants in general as well as in FSU immigrants [44,45,61,76,77]. In addition, FSU immigrants may experience difficulties associated with relocation and acculturation, despite high educational attainment and similarity among the racial majority of many settings [69,78,79,80,81]. For instance, even after controlling for education and other demographic factors, middle-aged FSU immigrant women in Israel were significantly less likely to be knowledgeable about heart disease than long-term Israeli Jews [82]. Cultural and family health beliefs, language, economic factors, and prior experiences with the native healthcare system among Hebrew-speaking FSU immigrant females were associated with less compliance with folic acid intake recommendations compared to Israeli Jews [83]. Whereas in the USA older FSU immigrant women with better acculturation and English language proficiency were more likely to participate in cancer screening [64]. In both the USA and Israel, FSU immigrants experienced challenges with healthy food choices due to lower acculturation and inability or lack of perceived need to read and understand food labels [84,85]. FSU immigrants in Germany reported to be dissatisfied with their medical care due to different cultural beliefs and health preferences and described communication issues with providers [86,87]. Acculturation and health beliefs influenced FSU immigrants’ preventative screening behaviors such that in Germany and America, they demonstrated decreased participation in health practices, lower medication adherence, and took a more passive approach to receive medical care than native-born populations [64,65,74,88,89]. Furthermore, likely similar to other immigrant communities, FSU immigrants may report limited trust toward health care providers and a healthcare system [90,91]. For example, older FSU immigrants preferred to use their traditional “tried and trusted” remedies and mistrusted media information [90]. This finding may explain why culturally sensitive educational materials were more useful, especially among low-acculturated FSU immigrants, in Germany [92]. Furthermore, learning the language of the host country and simultaneously maintaining Russian language proficiency posed challenges for FSU immigrants’ integration across countries [93]. The Russian language media may have the potential to effectively deliver health messages, especially to FSU immigrants with lower acculturation and lower proficiency in the host country’s language.

## 4. Discussion

Health literacy is still a relatively new concept (the majority of articles were published after 2000), which may explain the limited number of articles that met our inclusion criteria. However, it is an active research area. Currently, there are around 6000 health literacy articles listed under *Mesh Terms* on PubMed, a number of them are about immigrants, migrants, or refugees. However, FSU immigrants are underrepresented in health literacy research literature despite being one of the largest immigrant populations in the world. The six English language and one German language articles provided key insights to allow us to identify the specific needs of FSU immigrants from both individual and organizational perspectives and implications for future research, policy, and practice.

Future research on the health literacy of FSU immigrants should address health status, language proficiency, sociodemographic factors, health information sources, acculturation, the level of trust toward health providers and healthcare system, and patterns of utilization of health services to develop a greater understanding of personal health literacy among FSU immigrants. Older FSU immigrants and those with limited language proficiency of the host country, lower acculturation, and limited resources are likely to be the most vulnerable. In addition, males, more recent immigrants (2000s), and younger adult FSU immigrants warrant more scholarly attention as we found very little literature about them.

The quality, accessibility, and language of health materials are important factors in FSU immigrants’ ability to understand, evaluate, and apply health information. There is a need for culturally relevant and health literacy adopted educational materials for FSU immigrants, possibly distributed through their native language media, which is likely so for other immigrant communities. Culturally relevant information could include materials adopted not only to the immigrants’ cultural beliefs and health practices but also to the practices of their host countries so that immigrants can better understand how to navigate new healthcare systems and effectively utilize health services.

Only two studies assessed FSU immigrants’ health literacy, finding them to be lower than in native-born peoples and illuminating the need for more health literacy assessment and comparability of results across countries. Besides seeking a better understanding of factors contributing to FSU immigrant’s health literacy, we also recommend investigating health literacy across enabling and promoting factors. It appears that health organizations that adopt health literacy practices may help support FSU immigrants’ and other immigrants’ health promotion and disease management decisions. Strengthening organizational health literacy may empower and enable immigrants to find, understand, and use information and services to make health decisions and participate in care.

In addition, we found several articles on the topic of digital health literacy, specifically from Israel, which appears to be of growing interest and especially relevant with the emergence of the informational *infodemic* enhanced by the COVID-19 pandemic [24,94,95,96]. Digital health literacy among immigrants is of particular interest since immigrants often speak more than one language and are likely to search for information in several languages and across different countries impacting their health behaviors and decisions.

Global health literacy research is likely to be enhanced through multi-country collaborations. Our search in other languages was an important part of a comprehensive review of the topic as it led to a better-informed literature synthesis adding a global perspective on the topic. Searches in Russian, Hebrew, and German initiated a fruitful ongoing international collaboration such as joint publication, conference presentations, and other scholarly work. However, we also encountered challenges such as limited access to articles due to cost and procurement, unfamiliar databases, and lack of standardized terms making it difficult to identify and synthesize the literature. We warn researchers of the challenges related to inconsistency or recency of health literacy terms in non-English language literature and encourage more clarity and consistency in terminology across languages. Furthermore, as we found only one relevant article in a language other than English, we consider potential publication bias toward journals publishing in English [97]. There is possibly greater desirability or need to publish in English rather than Hebrew or German. For example, in Germany there are few journals dedicated to public health and migrant health; thus, researchers tend to publish in relevant English language journals. In Israel, publishing in the English language journals is considered to be more prestigious and is preferred as it allows researchers to reach a larger audience. In the Russian language, we found numerous articles (they were not about immigrants), but it is not clear if there is a need (publication silos) or access to disseminate findings to the English-speaking audiences. This trend may also be noted across research conferences at which presenters from non-English-speaking countries and with differing degrees of English proficiency often present in their non-native language and without interpreter support. Also, these language factors may influence care provision to immigrants as health providers whose primary language is not English may not have equal access to research publications. We believe that future research in the area of health and migration can and should be enhanced via international collaborations and multilingual reviews.

### Limitations

Despite the systematic approach to English language search, this review may not have captured all relevant studies. As described by inclusion and exclusion criteria, our review purposefully only included studies about FSU immigrants, migrants, or refugees that contained the exact term “health literacy” to avoid pulling in an overwhelming number of overlapping articles due to the ambiguity of health literacy related topics, which do not always use the term “health literacy” (e.g., use health compliance, health education). To address this gap, we synthesized articles that identified issues that fell within the scope of our interests but did not fit the precise guidelines of our search criteria (e.g., studies did not use the term “health literacy”). We also encountered difficulties searching databases in other languages, which could have prevented the identification of relevant articles. For example, databases and search engines adapted less well to languages other than English. In addition, despite health literacy being a common term in English, in other languages, it is a relatively new and less standardized term. Furthermore, it is possible we missed articles because we did not search health literacy by all 15 FSU republics in all databases.

This literature review was also limited by the general lack of articles that addressed health literacy issues in a specific population of Russian-speaking FSU immigrants. For various reasons, some immigrants from the FSU region may not speak or prefer not to speak Russian and may choose to dissociate from their region of origin. We noticed the need for more clarity in defining and distinguishing this population in research and quantifying the size of the Russian-speaking FSU diasporas in general. Considering that FSU, which consisted of numerous ethnic minorities and included many languages other than Russian, dissolved almost three decades ago, we also noticed that immigrants from FSU are described using different terms in the literature. For example, references may be based on the country of origin, region, language, religion, genetic makeup, historic circumstances (e.g., Ukraine, FSU, Russian, Jew, Slav, resettler). Also, we found articles that generalized potential FSU population without indicating specific characteristics (e.g., Eastern European), making it difficult to draw informative conclusions about what population was studied. Similarly, other immigrant diasporas who speak the same language but come from different cultures, historical and socioeconomic backgrounds (e.g., Spanish-speakers) may be aggregated. Thus, these limitations illuminated the need for well-defined study populations in research about immigrants in general and FSU immigrants specifically.

## 5. Conclusions

International migrants comprise 3.5% of the total world population, approaching 300 million people, of these, around 40 million live in Europe and 50 million in the USA [31]. Understanding and addressing the health and health literacy needs of immigrants is critical across countries. The importance of this topic is particularly relevant in the time of COVID-19 pandemic during which international movement has contributed to the spread of the disease, hampered containment, threatened the mitigation of outbreaks, and made the need for accessible, accurate, and easy to understand health information in different languages critical [21]. This multilingual integrative review examined and synthesized literature from several countries, which could be a useful model for other researchers.

Within the scope of health literacy literature generally and specifically around immigrants, it appears that FSU immigrants have been underrepresented. Despite the large and growing number of FSU immigrants worldwide, our understanding of their health literacy is limited. In support of personal health literacy, healthcare organizations, practitioners, policymakers, and stakeholders should support immigrants’ health by promoting ways and means to access care and maintain health in their host countries. Future research (including those conducted in languages other than English) should include a health literacy assessment, comparison across countries, identification of barriers, facilitators, and relevant predictors, and encourage the creation of culturally and linguistically appropriate materials to inform and support policies and health care provision for immigrants across countries.

## Figures and Tables

**Table 1:** Inclusion and exclusion criteria.

Category	Inclusion	Exclusion
Type of article	Qualitative and quantitative, peer-reviewed original research articles, editorials and think papers, empirical research, concept papers, reviews, surveys	Scale development studies, conference papers, research protocols, dissertations/theses
Population	Adult Russian-speaking immigrants or migrants or refugees from FSU	Children or adolescents, not clearly identified population
Article focus	Health literacy or health insurance literacy	Other
Language	English, German, Hebrew, Russian	Other

**Table 2:** Summary of supplemental literature searches.

Language	Search Strategy	Results
Russian	- Four databases and one search engine: Web of Science (Russian Science Citation Index), elibrary.ru, mediasphera.ru, cyberleninka.ru, yandex.ru; and, manual reference list review. - Searched up to 13 January 2019.- Search terms: медицинская грамoтнoсть OR здoрoвьесберегающее пoведение OR грамoтнoсть в вoпрoсах здoрoвья (all mean health literacy in Russian). - Two independent reviewers (U.K., P.P.) performed screening separately and met for consensus. A third reviewer (L.L.I) was available to resolve disagreements, but none occurred.	- A total of 257 unique articles were identified but no relevant articles were found at the title/abstract screening step. - We stopped our analysis at the title/abstract for the following reasons: (1) There is no concordance about health literacy terminology in the Russian language literature with more than eight circulating terms, making it difficult to identify and synthesize literature; and, (2) only recently a concordant term “грамoтнoсть в вoпрoсах здoрoвья”, was proposed by Syrtsova et al. (2016) and later accepted by the World Health Organization [51]. - All articles were published in the 2000s. - We found no articles about FSU immigrants (those who moved to another country outside of the FSU region).
German	- Google Scholar and manual reference list search. - Searched up to 27 October 2020. - Search terms: Gesundheitskompetenz (health literacy) AND (Sowjet* OR Soviet OR russischsprachig OR Russland OR russisch*) AND (Migranten OR Menschen mit Migrationshintergrund OR Menschen mit Zuwanderungsgeschichte OR Flüchtling OR Geflüchtete OR Aussiedler). - Included articles, book chapters, and reports. - Also searched by countries of origin. - Search and review performed by E.-M.B.	- A total of 62 unique articles were identified and screened at full text. - One article by Horn et al. (2015) met the inclusion criteria [52]. - No articles assessed health literacy. - Identified publications were either about immigrants in general or not specifically about health literacy in FSU immigrants or health literacy was mentioned as an explanatory factor for differences in health status or health service utilization but was not the focal topic. - No articles identified when searched for health literacy by FSU region countries. - The term health literacy is still relatively new in Germany.
Hebrew	- Google Scholar and University of Haifa library. - Searched up to 28 October 2020. - Search terms: אוריינות בריאות (health literacy) ANDרוסית (Russian) - Search and review performed by D.E.K.	- A total of 5 unique articles were identified when searching for both terms. - For reference, a search for just “health literacy,” without specifying “Russian,” yielded a total of 54 unique articles. - Identified articles did not include health literacy as a focal topic. - A few older articles (from the 1990s) examined (either exclusively or as part of a larger study) the use of health services by the Russian-speaking FSU immigrants. - The term health literacy is still relatively new in Israel.

**Table 3:** Summary of studies.

Author & Year	Country (State)	Purpose	Setting	Sample	Type	Findings	Theoretical Framework or Model	Discipline	Evaluation
English Language									
Kostareva et al. 2020	USA, Israel, Germany, Kazakhstan, Russia	To provide an overview of FSU immigrants’ background and discuss system-level factors relevant to FSU immigrants’ health literacy by looking at post-Soviet healthcare systems (Russia, Kazakhstan) and the healthcare systems of top host countries (USA, Israel, Germany)	Conceptual paper; cross-country comparison	N/A	Scoping review	Multiple factors such as FSU immigrants’ health and cultural beliefs, previous experiences with and exposures to their native healthcare system may influence their health literacy in host countries	Sorensen’s Integrated Health Literacy Model	Nursing and public health	1
Nakash et al. 2020	Israel	To examine the association between mental health literacy, emotional distress, and the role of immigration status among older adults	Self-reported questionnaires in Russian; participants recruited online and through social clubs	222 Russian-speaking FSU immigrants (mean age 70)	Quantitative (assessed with Mental health literacy scale)	FSU demonstrated significantly lower mental health literacy and higher emotional distress	None	Social work and psychology	2
Levin-Zamir et al. 2016	Israel	To examine the relationship between health literacy, health behavior, sociodemographic indicators, and self-assessed health in adults	Face-to-face home interviews in Russian	55 Russian-speaking FSU immigrants (mean age 50)	Quantitative (assessed with HLS-EU-Q16	FSU demonstrated inadequate health literacy and long-term Israeli Jews sufficient health literacy but no significant difference	None	Public health	2
Van Son 2014	USA (Washington)	To develop and test 12 culturally appropriate dietary and physical activity education materials to improve health literacy and manage diabetes	Focus group (n = 10, age 65+) in Russian and telephone survey (n = 14)	24 Russian-speaking Slavic immigrants	Qualitative	Culturally relevant to FSU educational materials around diabetes uploaded online and demonstrated to be of demand	The Plate Model and the Physical Activity Pyramid	Nursing	1
Bailey et al. 2012	USA (San Francisco and Chicago)	To evaluate the efficacy of health literacy informed, translated drug label instructions in comparison to standard instructions	Clinics and community-based organizations for low-income urban populations	40 Russian-speakers with limited English proficiency (mean age 64)	Quantitative	Translated, health literacy adopted drug labels led to significantly greater understanding, regimen dosing, and regimen consolidation comparing to standard instructions	None	Medicine	2
Sullivan 2009	USA (Alaska)	To describe a teaching-learning strategy emphasizing community partnership between nursing students, an immigrant refugee program, and a community literacy program	Immigrant-refugee and community literacy programs	36 Russian refugees	Qualitative	Russian-speaking refugees’ top 3 health concerns: healthy eating, women’s health, and high blood pressure	Leininger’s Culture Care Theory	Nursing	1
German Language									
Horn et al. 2015	Germany	To identify factors relevant to health literacy in the context of health counseling among migrants	Interviews at a patient health counseling organization with health consultants (experts in Turkish and Russian health counseling) and users of health counseling	6 focus groups, 24 interviews with health consultants, 9 potential and 9 actual users of counseling of Russian and Turkish background	Qualitative	Russian-speaking FSU immigrants appear to have difficulty understanding the German healthcare system and utilizing health services effectively	Sorensen’s Integrated Health Literacy Model	Public health	1
